# Royal Jelly and Fermented Soy Extracts—A Holistic Approach to Menopausal Symptoms That Increase the Quality of Life in Pre- and Post-menopausal Women: An Observational Study

**DOI:** 10.3390/nu16050649

**Published:** 2024-02-26

**Authors:** Andreea Balan, Marius Alexandru Moga, Andrea Elena Neculau, Maria Mitrica, Liliana Rogozea, Petru Ifteni, Lorena Dima

**Affiliations:** 1 Department of Fundamental, Prophylactic and Clinical Sciences, Faculty of Medicine, Transilvania University of Brasov, 500019 Brașov, Romania; andreea.balan@unitbv.ro (A.B.); r_liliana@unitbv.ro (L.R.); petru.ifteni@unitbv.ro (P.I.); lorena.dima@unitbv.ro (L.D.); 2 Department of Medical and Surgical Specialties, Faculty of Medicine, Transylvania University of Brasov, 500019 Brașov, Romania; mogas@unitbv.ro (M.A.M.); mitrica.maria@unitbv.ro (M.M.)

**Keywords:** royal jelly, menopause, fermented soy extracts, vasomotor symptoms

## Abstract

Background: The objective of this study was to determine the effects of royal jelly and fermented soy extracts on menopausal symptoms and on quality of life in pre- and post-menopausal women. Materials and method: This prospective observational study was carried out in a Clinical Hospital of Brasov, Romania, during June 2020 and December 2021. Eighty pre- and post-menopausal women, aged between 45 and 60 years, were included in two groups. The first group (40 women) received a dietary supplement with fermented soy extract twice a day for eight weeks and the second group (40 women) received the same dietary supplement with fermented soy extracts and 1500 mg of royal jelly capsules for eight weeks. After the treatment, the MENQOL score, DASS-21 score, and the mean number and intensity of daily hot flushes were recorded and compared with baseline values. Results: After eight weeks of treatment, the score of the MENQOL questionnaire and all its domains’ scores decreased in comparison with the baseline in both groups (*p* < 0.001). Also, the DASS-21 score (*p* < 0.001), depression score (*p* < 0.001), anxiety score (*p* < 0.001), and stress score (*p* < 0.001) improved. The mean number and the intensity of hot flushes decreased in both groups (*p* < 0.001). Comparing these variables after the treatment in both groups, we observed that the women who received dietary supplements with fermented soy extracts and royal jelly capsules recorded better scores for MENQOL (vasomotor, physical, and psychosocial domains) and a more reduced mean number of daily hot flushes. Conclusions: This observational study suggests that both dietary fermented soy supplements and royal jelly capsules possess beneficial effects against menopausal symptoms, increase the quality of life in pre- and post-menopausal women, and that the effects might be significantly improved if those dietary supplements are administered in association.

## 1. Introduction

Menopause is the result of the permanent loss of cyclic ovarian activity and it is considered that a woman spends approximately one-third of her lifetime in the post-menopausal period [1]. Menopausal transition is defined as the time preceding the normal menopause, during which the declining ovarian function causes various disturbances to the menstrual cycles. Moreover, a number of specific clinical, biological, and, of course, endocrinological changes characterize this transition to menopause [2].

According to the previous published data, the age at which women enter menopause has a high degree of variability, depending on many genetic, environmental, or lifestyle factors. This age has been established to be between 45.5 and 47.5 years old [3]. For Caucasian women, the average age for menopause is between 50 and 51–55 years, while for women from non-industrialized countries and various ethnic groups, the data are not reliable [4]. According to Sallam et al. [5], the mean age of menopause in Egypt is 46.7 years, which is low in comparison with that of the Western population. Moreover, the attitude towards this novel biological state is more positive in Western women compared with that in other ethnic groups, including the Egyptian population, which has reported a greater incidence of menopause-related symptoms.

During menopause, the endocrinological panel of the women is dominated by sudden estrogen withdrawal [6]. The decrease in estrogen levels induces a wide range of menopause-related symptoms that produce different degrees of life disruption and may greatly affect the quality of life [7]. Quality of life is a multidimensional concept, which lacks a precise definition. The World Health Organization (WHO) has defined this concept as the perception of people about their position in life in the context of the culture and value systems and in relation to their goals, standards, and expectations [8]. Menopausal symptoms tend to decrease quality of life in midlife women, and the study of this aspect has become a very important part of modern clinical practice. Of course, individual perceptions about the quality of life during this period might be influenced by different socio-cultural factors.

During this period, women experience many symptoms, such as hot flushes, sleep disturbances, sexual impairment, vaginal dryness, depression, anxiety, insomnia, bone and joint complaints, headaches, and a reduction in muscle mass [9]. Vasomotor symptoms, such as hot flushes or night sweats, have been reported as the most debilitating symptoms, and they are secondary to the changes in sex hormone levels [10]. Psychological symptoms frequently occur and, depending on their severity, may require specialized treatment. These symptoms consist of depressive episodes, anxiety, irritability, decreased concentration, or memory loss. The sexual life of menopausal women is also affected by a combination of physical (genitourinary syndrome and vaginal dryness) and psychological factors. Many women reported decreased sexual desire during the menopausal transition or in the postmenopausal period, which negatively affects their relationships [11].

Conventionally, hormone replacement therapy has been used for decades by the majority of women to treat all these menopause-related symptoms. However, the adverse effects of this therapy, such as abnormal uterine bleeding, breast cancer, or breast tenderness, sometimes outweigh the benefits [12,13]. For this reason, alternative medicine, which includes natural and non-hormonal products, such as food supplements, herbal products, or phytoestrogens that could promote healthy aging and extended lifespan, has gained more and more trust from patients and consumption rates are constantly rising. The WHO reported that approximately 85% of the population in developing countries uses plants or herbal products to treat a wide range of medical conditions [14]. Approximately 52% of the women aged 55 years or older, married, and with a high level of education, with chronic illnesses, such as diabetes or high blood pressure, reported the consumption of phytotherapeutic compounds for the alleviation of menopausal symptoms [15]. However, most women using alternative medicine for menopausal syndrome do not inform their physicians [16].

Soybeans represent one of the most widespread natural alternatives to hormone replacement therapy due to their increased content of isoflavones, which are a class of phytoestrogens found in a variety of plants [17]. Isoflavones are phenolic compounds that are able to exert estrogen-like activities by binding estrogen receptors (ERs), including ERα, which is mainly distributed in the breast and uterus, and Erβ, which predominates the cardiovascular and urogenital systems and the bones [16].

Soy contains twelve different types of phytoestrogens. Also, it contains three types of isoflavones in four chemical forms. Genistein, daidzein, and glycitein are the aglycones. They can form three glucoside forms: the β-glucosides daidzin, genistin, and glycitin, the 6″-O-malonyl-glucosides, and the 6″-O-acetyl-glucosides conjugate [18,19]. Aglycones are known to be more bioavailable than glycosides [20].

Isoflavones mainly occur in soybeans in the form of biologically inactive glycosides, like genistin and daidzein. After ingestion, these molecules are hydrolyzed in the gut by intestinal bacterial β-glucosidases, resulting in the formation of appropriate bioactive aglycones: genistein and daidzein [21]. Daidzein can be metabolized to dihydrodaidzein and then to S-equol, which has a preference for binding to ERβ [22], or O-desmethylangolensin [23]. Meanwhile, genistein can be hydrolyzed to dihydrogenistein and then to 6ʹ-hydroxy-O-desmethylangolensin, which, in turn, can be degraded to p-ethylphenol [24]. Daidzein has a proven higher bioavailability than genistein because it has a longer half-life in the intestine [25]. Therefore, the fermentation process of soybeans increases the content of the more bioavailable aglycones [21]. During the fermentation process, the aglycone contents increase owing to increased β-glucosidase activity, whereas the contents of glycoside forms decrease [26]. In fermented soy products or dietary supplements, the aglycone values may vary from 40 to 100% [27].

Considering the estrogen-like actions of isoflavones, epidemiological studies have explored the connection between the consumption of soybeans and the alleviation of menopausal syndrome [28], but their efficiency is still highly debated. Considering the great amount of metabolic activities and biotransformations that take place during the soybean fermentation process, the use of products or dietary supplements based on fermented soy extracts for the management of menopausal symptoms could represent a much better choice than the use of isoflavones.

Royal jelly (RJ) is a yellowish and creamy substance secreted by the mandibular and hypopharyngeal glands of nurse bees. It is known as a “superfood” due to its multiple biological effects, and it is used to feed queen bees throughout their lifetime and worker bees during the larval stage [29]. Recent reports have shown that RJ possesses estrogen-like properties, and it can be used as a valuable medicinal agent for healthy aging and longevity [30] and for the alleviation of postmenopausal symptoms. Moreover, RJ possesses anti-inflammatory, antibacterial, antioxidative, vasodilative, and anticancer effects [31].

The chemical composition of RJ mainly depends on the geographic area and the seasonal conditions. This substance contains 50–60% water, a great amount of proteins and peptides (major royal jelly proteins MRJP1–MRJP9), amino acids, sugars (15% of the total composition), lipids and fatty acids (10-hydroxy-2-decenoic acid, sebacic acid, and 10-hydroxydecanoic acid), vitamins, minerals, phenols, flavonoids, and hormones [32,33]. The first clinical trial regarding the efficiency of RJ supplementation during menopause was conducted by Asama et al. [34] in the year 2018. Their results showed that 800 mg of RJ/day decreased anxiety, backache, and lower back pain in a population of postmenopausal Japanese women. Also, the safety of RJ supplementation has been confirmed by some studies [35].

The aim of our study was to evaluate the effects of a dietary supplement containing fermented soy (FS) extracts and the effects of these supplements associated with RJ capsules on menopausal symptoms and on the quality of life of pre- and post-menopausal women in a clinical setting. To our knowledge, no study analyzing the cumulative effects of fermented soy extracts and RJ on menopausal symptoms has been previously published.

## 2. Materials and Methods

### 2.1. Participants and Methodology

This observational prospective study was conducted in the Clinical Hospital of Obstetrics and Gynecology “Dr. I.A. Sbarcea” of Brasov, Romania, during June 2020 and December 2021, according to the guidelines of the Declaration of Helsinki. It was approved by the Ethics Committee of the Clinical Hospital of Obstetrics and Gynecology “Dr. I.A. Sbarcea” of Brasov, Romania (5080/16 June 2020) and also by the Ethics Committee of Transilvania University of Brasov. This study included 97 women in peri- and post-menopause from Brasov County and the surrounding areas, who presented to the hospital ambulatory for menopausal symptoms and were treated either with a dietary FS supplement or a mixture of this dietary supplement and RJ capsules. As this is an observational study, the choice of therapy was entirely up to the individual physicians and patients. In order to obtain accurate results, all the patients included in this study received the same products: a dietary supplement with 322 mg of FS/capsule (Femarelle Recharge^®^, Se-cure Pharmaceuticals Ltd., Airport City, Israel), two capsules per day, and the same dietary FS supplement associated with RJ capsules (Parapharm) containing each 500 mg of freeze-dried royal jelly, divided into three daily doses (1500 mg/day).

**Figure 1:**
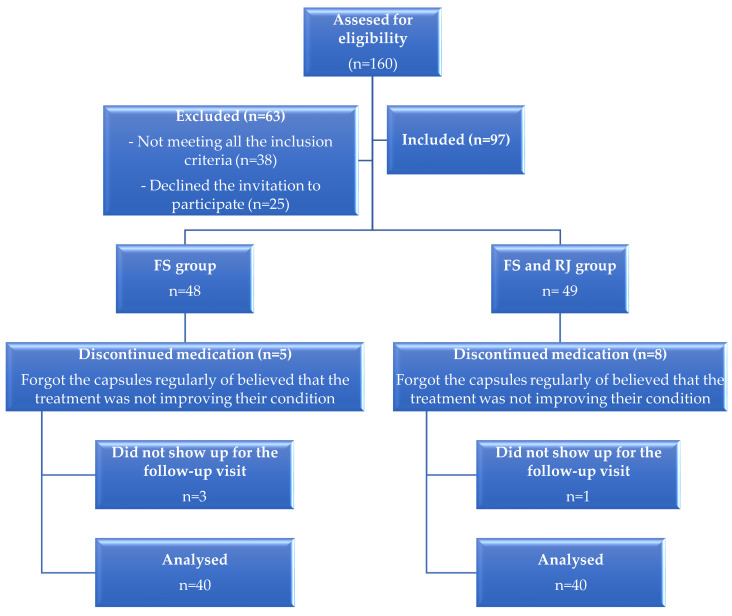
Diagram of the groups’ enrollment, follow up, and final analysis.

One hundred and sixty peri- and post-menopausal women were invited to participate in this study, but 38 of them did not meet all the eligibility criteria, 25 declined the invitation to participate in this study, 13 patients discontinued their medication during the treatment period or the administration was inappropriate, and 4 did not show up for the post-treatment visit. Forty-eight women were initially included in the FS group, and forty-nine women were included in the FS and RJ group. From these, only 40 patients from each group were considered for the final statistical analysis (see Figure 1).

The inclusion criteria were:
  - Mentally oriented women with menopausal symptoms (at least 3 hot flushes per day);
  - Aged between 45 and 60 years;
  - Able to read and write;
  - Women free from any chronic treatments;
  - Women who have received the above-mentioned indications of treatment from their physician for the management of menopausal symptoms.

The exclusion criteria were:
  - Women who received hormonal replacement therapy (HRT) or any other naturopathic treatments for the alleviation of menopause-related symptoms;
  - Women who received other dietary supplements based on soy extracts or different doses of RJ;
  - Women with chronic diseases (neoplasia, diabetes mellitus, and chronic hypertension);
  - Women with psychiatric pathology;
  - Women who refused to be included in this study.

All the patients were recruited from the ambulatory of the hospital. The researchers briefly explained the purpose of this study to the approached women who met the inclusion criteria. All the women were informed that their participation would be voluntary and that they would not be remunerated if they were to be included. All the participants signed an informed consent before being included in this study and approved the statistical analyses of the data and the publication of the results. This study’s design involved two visits: before the beginning of the treatment (at baseline) and after 8 weeks of treatment. During this period, the patients were closely monitored for adverse effects of the treatment.

At baseline, after the acceptance of the participants was obtained, general characteristics and anamnesis were collected. Afterward, they were asked to complete an interviewing sheet, the menopause-specific quality of life questionnaire (MENQOL), and the Depression, Anxiety and Stress Scale 21 (DASS-21). Approximately 40–60 min was allocated to each woman. Each patient personally performed the completion of the questionnaires in the presence of an investigator, but without them influencing the answers. The second visit, after 8 weeks of treatment, was similar to the baseline visit.

### 2.2. Instruments

Three tools were used in order to collect the data: an interviewing sheet, the MENQOL, and the DASS-21R scale. The interviewing sheet was designed by the authors and included the following information: age, residence, menopausal stage (menopausal transition or post-menopause), type of menopause (natural or surgically induced), body mass index (BMI), physical exercises, alimentation characteristics, and daily number and intensity of hot flushes (0—extremely weak, 1—very weak, 2—weak, 3—mild, 4—intense, 5—very intense, and 6—extremely intense). These instruments were first piloted on 10 women in order to determine their comprehensibility and ease of use. All these women were excluded from the final cohort.

The second tool was represented by the MENQOL questionnaire. The patients received a Romanian translated version of this questionnaire, developed by the authors with a license received from Mapi Research Trust. This is a self-report measure assessing the presence and severity of menopausal symptoms, first designed by Lewis J. and Hilditch J.R. [36]. The main goal of the authors was to develop a valid quality-of-life questionnaire during the menopausal period, based on the women’s self-reported experience.

The MENQOL questionnaire contains 29 items divided into four domains, as follows: vasomotor, psychosocial, physical, and sexual domains, each of them having a different number of items. In order to obtain a total MENQOL score, a seven-point Likert scale applied by the patient for each item was converted to an eight-point scale, ranging from 1 to 8. If the woman had not experienced the item in the last month and she responded with “no”, then the score was “one”. If the woman had experienced the symptom, but it was not at all bothersome (“0”), the score was “two”. The scores between “three” and “eight” corresponded to symptom intensities ranging from “1” to “6” and indicated increasing levels of bother experienced from this symptom. Each domain obtained a final score, which was the mean of the converted item scores forming that domain. We considered a score ranging between 1 and 4 as mild, between 5 and 6 as moderate, and between 7 and 8 as severe. The best reliability of data was established for women aged between 41 and 62 years who were 1 to 9 years after their last menses [37]. Cronbach’s alpha or coefficient alpha were used in order to determine if the multiple-question Likert scale surveys were reliable or consistent. Excellent internal consistency is defined by a Cronbach’s alpha value greater than 0.9, while a Cronbach’s alpha value smaller than 0.5 suggests unacceptable internal consistency [38]. In our study, the Cronbach’s alpha value was 0.71, indicating acceptable reliability.

The Depression Anxiety and Stress Scales is a self-report instrument designed to measure the negative emotional states of depression, anxiety, and stress. It is based on the concept that differences between depression, anxiety, and stress in normal subjects are essentially differences of degree. These scales do not allow the inclusion of the patients to various diagnostic categories postulated in the Diagnostic and Statistical Manual of Mental Disorders, because DASS-21 is not a diagnostic tool. It is mainly based on a dimensional, rather than a categorical, conception of psychological disorder [39]. The DASS-21 scale is a short version of the original DASS index, which consists of 42 questions. DASS-21 includes 3 subscales: depression, anxiety, and stress. The participants of our study were asked to use a 4-point severity scale for each symptom experienced in the past week (from 0, which meant that the woman had “never experienced” the symptom, to 4—the woman experienced the symptom “most of the time” in the last week). The total scores range from 0–28+ for the depression subscale, 0–20+ for the anxiety subscale, and 0–34+ for the stress subscale [40]. The depression dimension assesses lack of interest, anhedonia, hopelessness, self-deprecation, and devaluation of life. The anxiety subscale assesses situational anxiety, autonomic stimulation, and the subjective experience of anxious affect. Last, but not least, the stress dimension assesses relaxation difficulties, impatience, irritability, and the tendency to be over-reactive and easily upset [41].

All the participants completed the Romanian validated version of the DASS-21, known as DASS-21R. The internal consistency was explored for each subscale of the DASS-21, and the Cronbach’s alpha coefficients were adequate: depression (0.79), anxiety (0.82), and stress (0.80).

### 2.3. Statistical Analyses

Version 26 of Statistical Product and Service Solutions software (IBM SPSS) was used for the statistical analysis of the data. The normality of the distributions of continuous variables was tested using the Kolmogorov–Smirnov test, while the equality of variances was tested using Levene’s test.

The *t* test for independent samples was used in order to compare the means according to dichotomous variables. Also, we used the t-paired test in order to compare means between paired samples. A value of the statistical significance coefficient *p* less than 0.05 was considered significant. Furthermore, when the *p* values were less than or equal to 0.01 they were considered highly significant.

## 3. Results

A total of 80 patients, 40 in the FS group and 40 in the FS and RJ group, finished the study and were considered for the statistical analyses. The general characteristics of the analyzed population are shown in Table 1, and no statistically significant difference was present in terms of age, residence, menopausal stage, type of menopause, BMI, physical activity, or alimentation particularities.

The baseline characteristics of the two groups were also comparable and no statistically significant difference was present. The baseline MENQOL scores and the scores of its four domains, DASS-21 scores, depression subscale scores, anxiety subscale scores, and stress subscale scores, and the mean number and the intensity of hot flushes are presented in Table 2.

After eight weeks of treatment, in the FS group, we observed a statistically significant decrease in the mean number and intensity of hot flushes (*p* < 0.001). The mean number of hot flushes decreased from 8.7 (SD 3.82) to 4.53 (SD 3.79). Considering the MENQOL questionnaire, the mean changes from baseline showed a favorable effect of the dietary supplement with fermented soy extracts. The mean scores of the vasomotor, psychosocial, physical, and sexual domains also improved in comparison with the mean baseline values. Furthermore, the consumption of this dietary supplement improved the mean DASS-21 scores and the mean scores of all its subscales (depression, anxiety, and stress). The MENQOL scores, DASS-21 scores, and vasomotor symptoms in the FS group at baseline and at the eight-week visit are illustrated in Table 3.

In the second study group, who received the same dietary supplement with FS in association with RJ capsules, we also observed a statistically significant improvement in the mean number (from 7.18 (SD 2.18) to 2.85 (SD 1.27)) and intensity of hot flushes (*p* < 0.001). The MENQOL and DASS-21 scores also exhibited a significant decrease compared with the baseline values (Table 4).

Beneficial effects were recorded for both groups after eight weeks of treatment compared with the baseline. However, we compared the vasomotor symptoms, MENQOL, and DASS-21 scores after treatment in both groups. We observed that the MENQOL score (*p*= 0.008), vasomotor (*p* = 0.001), psychosocial (*p* = 0.026), and physical domain (*p* = 0.041) scores were statistically significantly lower in the FS and RJ group than in the FS group. Furthermore, the women who received both supplements recorded a more decreased mean number of hot flushes (4.53 (SD 3.79) vs. 2.85 (SD 1.27)). The comparison of these variables after eight weeks of treatment in both groups is presented in Table 5.

## 4. Discussion

In this study, eight weeks of supplementation with FS extracts or FS extracts plus RJ capsules was effective in reducing the mean number and intensity of daily hot flushes in peri- and post-menopausal women. Moreover, statistically significant changes in the quality of life scores were observed in all the MENQOL domains in both study groups. The scores of the DASS-21 scale were also improved in both groups after eight weeks of daily administration of these dietary supplements.

To date, we have not found in the literature a previous study on the cumulative effects of dietary FS extract supplements and RJ on menopausal symptoms. However, several studies, which we will briefly present below, on the effects of soybean extracts (isoflavones) or RJ on menopausal symptoms have been published. Our results are consistent with those reported in some studies, but not in others. This inconsistency could be explained by the different doses and periods of administration. Moreover, the observational versus the randomized study designs should be considered.

Chedraui et al. [42] performed a study on 50 symptomatic climacteric women who received 100 mg of soy-derived isoflavones daily for 3 months. The authors evaluated the frequency and intensity of hot flushes, menopausal symptoms (Menopause Rating Scale), and mood using the Hamilton Depressive Rating Scale (HDRS). After the treatment, the authors observed that the number and intensity of hot flushes significantly decreased. Moreover, the menopausal symptoms and total HDRS scores decreased in comparison with the baseline.

Another randomized, placebo-controlled trial was performed by Amato et al. [43] in order to assess the effect of 80 mg or 120 mg of soy isoflavone oral supplementation on the quality of life of post-menopausal volunteers. According to their results, the MENQOL scores at 12 and 24 months were similar to those at the baseline, and no difference in the domains’ scores was noticed among the two groups. Ho et al. [44] also investigated whether the oral intake of 80 mg soy-derived isoflavones for 6 months improved quality of life in Chinese postmenopausal women and cognitive function. In comparison with the placebo, the administration of this dietary supplement did not improve the MENQOL scores, nor the cognitive function of post-menopausal women.

Another study that reported that soy isoflavone supplements did not improve the overall quality of life and climacteric symptoms in post-menopausal women was conducted by Lee et al. [45]. Eighty-seven post-menopausal women received 70 mg/day of isoflavones or a placebo for 12 weeks. The MENQOL scores after the treatment were not different to the baseline.

Over 50 trials have investigated the efficacy of isoflavone-rich products on menopausal symptoms, and the results remain controversial [46]. S-equol, which is converted by the intestinal bacteria from daidzein, is the isoflavone-derived metabolite with the greatest estrogenic and antioxidant activity. S-equol has been proposed to have superior health benefits to those of its parent isoflavone [47]. The process of whole soy germ fermentation with a strain of equol-producing lactic acid bacteria develops a S-equol-containing supplement [48]. A double-blind, placebo-controlled trial that included postmenopausal Japanese women, randomly assigned to consume a placebo or 10 mg/day of S-equol-containing supplement for 12 weeks, reported a great decrease in the number and severity of hot flushes from the baseline in the S-equol group [49]. The results of our study could be consistent with the results of this study, because our volunteers also consumed a S-equol-containing supplement. However, it is difficult to interpret the results due to the different doses and periods of administration. Also, the placebo effect observed in studies on hot flushes could complicate the interpretation of the results.

DT56a is a natural medication that contains a variety of phytoestrogens derived from tofu. A study conducted by Sanchez-Borrego and collab. [50] in 2015 included 631 Spanish menopausal or peri-menopausal women who received this dietary supplement for 4 weeks in order to analyze its efficacy in the reduction of menopausal symptoms. After this period, 80.7% of the patients reported that their hot flushes were ‘better’ or ‘much better’. The patients included in our study, in both cohorts, received the same commercial product as the women included in this study and our results also showed a significant reduction in the number and intensity of hot flushes in post- and peri-menopausal women.

In addition to a wide range of beneficial health effects, it has been proven that RJ from honeybees possesses estrogenic activity and it might be used for the management of menopausal symptoms. In the present study, we observed that women who received a dietary supplement with FS extracts associated with RJ capsules for 8 weeks reported a decrease in the mean number and intensity of hot flushes. Also, the MENQOL scores and DASS-21 scores were improved compared with the baseline values. In comparison with the women included in the FS group, those who also received RJ capsules had a more significant decrease in the scores for the vasomotor, psychosocial, and physical domains and the total MENQOL scores were more decreased. Moreover, these women also reported fewer daily hot flushes than those included in the FS group. The differences between groups in terms of hot flushes’ intensity were not significant.

Our results are mainly consistent with those reported in other studies regarding the effects of RJ on menopausal symptoms. However, the differences between doses, periods of administration, and study design make these results difficult to interpret. Sharif et al. [51] conducted a double-blind randomized clinical trial in order to assess the effects of 1000 mg/day of RJ on menopausal symptoms in 200 post-menopausal women. After 8 weeks of daily administration, the menopausal score significantly reduced in the study group in comparison with the placebo group.

Asama et al. [34] demonstrated that oral treatment with 800 mg/day of RJ for 12 weeks significantly decreased the anxiety score in post-menopausal women. In our study, the anxiety subscale score was more decreased in the FS and RJ group than the baseline values but the differences between groups were not statistically significant after 8 weeks of treatment.

In 2018, a group of medical researchers from Japan’s Kindai University included 42 post-menopausal women in a study in order to assess the effects of 800 mg/day of RJ on menopausal symptoms. Their results indicated that after only 4 weeks of treatment, menopausal symptoms significantly improved: hot flushes, sweating, dizziness, and physical scores decreased, and anxiety and depressive moods were also reduced [52].

Regarding the adverse effects, in the FS group, one patient reported an increase in hot flushes intensity during the treatment and two women reported isolated headaches after the ingestion of the dietary supplement. In the FS and RJ group, one woman reported a transitory skin rash. However, the observational design of this study and the inability of the authors to manage the treatment response could also have affected the reporting of adverse effects by patients.

## 5. Conclusions

In conclusion, this prospective observational study suggests a possible improvement in the quality of life and vasomotor symptoms in menopausal women who received dietary supplements based on FS extracts, associated with RJ capsules or not. Furthermore, the negative emotional states of depression, anxiety, and stress recorded improvements after 8 weeks of treatment, either with FS extracts or RJ capsules and FS.

The association between these two dietary supplements was superior in terms of mean MENQOL scores and vasomotor, psychosocial, and physical domain scores. Also, the patients who received both dietary supplements reported a more significant decrease in the mean number of hot flushes.

Therefore, the association between these two natural products might represent an interesting alternative for the alleviation of menopausal symptoms and for increasing quality of life in women who reject conventional hormone therapy. Their low price, their beneficial effects, and safety, at least in the medium term, could transform these agents into a valuable alternative for the management of menopause-related symptoms, which, in recent years, have become a public health problem that affects the lives of thousands of women.

## 6. Limitations of the Study

The small number of participants represents the major limitation of this study, in our opinion. The limited activity of the hospital, imposed by the COVID-19 pandemic, was reflected in the number of women included in this research. In addition, their fear of getting sick reduced the number of hospital visits.

Another limitation of this study is that a main part of the database was represented by self-reported data. The way in which the participants perceived their menopausal symptoms and their quality of life may have been different from how health professionals would have perceived them, and this perception could have influenced the results. Also, the observational design of this study could be considered a limitation, because the authors had no access to the treatment, and they could not manage the treatment response or the adverse effects of the therapy. Moreover, this study did not include a placebo and was not a double-blind study.

## Figures and Tables

**Table 1:** General characteristics of the women enrolled in the observational study.

Variable	FS Group (n.: 40) ± SD	FS and RJ Group (n.: 40) ± SD	*p*
Age (years)	50.67 ± 3.48	50 ± 3.47	0.267
Residence			
Rural	14 (35%)	14 (35%)	0.875
Urban	26 (65%)	26 (65%)	
Menopausal stage			
Menopausal transition	14 (35%)	17 (42.5%)	0.362
Post-menopause	26 (65%)	23 (57.5%)	
Type of menopause			
Natural menopause	33 (82.5%)	32 (80%)	0.346
Surgically induced menopause	7 (17.5%)	8 (20%)	
BMI (body mass index) (kg/m^2^)	28.54 ± 5.54	28.21 ± 5.31	0.953
Normal weight	13 (32.5%)	13 (32.5%)	0.656
Overweight	14 (35.0%)	18 (45.0%)	
Grade I obesity	9 (22.5%)	6 (15.0%)	
Grade II obesity	1 (2.5%)	0 (0.0%)	
Grade III obesity	3 (7.5%)	3 (7.5%)	
Physical activity			
No physical activity	36 (90.0%)	30 (75.0%)	0.205
>3 times/week	0 (0.0%)	0 (0.0%)	
1–3 times/week	2 (5.0%)	6 (15.0%)	
1–3 times/month	2 (5.0%)	4 (10.0%)	
Alimentation			
Normal diet	32 (82.5%)	32 (82.5%)	0.885
Vegetarian diet	3 (7.5%)	4 (10.0%)	
Diet based on dairy, eggs, and vegetables	4 (10.0%)	2 (5.0%)	
Dairy-free diet	0 (0.0%)	1 (2.5%)	

**Table 2:** Baseline characteristics of the two groups.

Variable	FS Group (±SD)	FS and RJ Group (±SD)	*p*
MENQOL score	113.53 ± 39.05	111.30 ± 26.17	0.766
Vasomotor domain	16.83 ± 3.19	17.13 ± 5.67	0.724
Psychosocial domain	26.93 ± 10.00	26.98 ± 6.52	0.979
Physical domain	54.68 ± 18.07	53.20 ± 17.25	0.746
Sexual domain	15.35 ± 6.27	14.00 ± 5.93	0.326
DASS-21 score	17.50 ± 11.81	20.43 ± 10.70	0.249
Depression score	5.55 ± 4.18	6.33 ± 3.50	0.372
Anxiety score	6.30 ± 5.20	6.60 ± 3.76	0.768
Stress score	5.65 ± 3.74	7.50 ± 5.59	0.086
Hot flushes (n./day)	8.75 ± 3.82	7.18 ± 2.18	0.189
Hot flushes intensity			
Extremely weak (0)	0 (0.0%)	0 (0.0%)	0.529
Very weak (1)	0 (0.0%)	0 (0.0%)	
Weak (2)	0 (0.0%)	1 (2.5%)	
Mild (3)	17 (42.5%)	8 (20.0%)	
Intense (4)	13 (32.5%)	13 (32.5%)	
Very intense (5)	4 (10.0%)	15 (37.5%)	
Extremely intense (6)	6 (15.0%)	3 (7.5%)	

**Table 3:** MENQOL scores, DASS-21 scores, and vasomotor symptoms in FS group at baseline and at the follow-up visit.

Variable	Baseline	Follow-Up Visit	*p*
MENQOL score	113.53 ± 39.05	100.53 ± 38.56	<0.001
Vasomotor domain	16.83 ± 4.30	11.25 ± 4.53	<0.001
Psychosocial domain	26.93 ± 10.09	22.60 ± 9.25	<0.001
Physical domain	54.68 ± 22.91	50.05 ± 22.50	<0.001
Sexual domain	15.35 ± 6.27	14.75 ± 5.87	<0.001
DASS-21 score	17.50 ± 11.81	14.05 ± 11.23	<0.001
Depression score	5.55 ± 4.18	4.45 ± 3.96	<0.001
Anxiety score	6.30 ± 5.20	4.72 ± 4.97	<0.001
Stress score	5.65 ± 3.73	4.88 ± 3.43	<0.001
Hot flushes (n./day)	8.75 ± 3.82	4.53 ± 3.79	<0.001
Hot flushes intensity			
Extremely weak (0)	0 (0.0%)	2 (5.0%)	<0.001
Very weak (1)	0 (0.0%)	7 (17.5%)	
Weak (2)	0 (0.0%)	12 (30.0%)	
Mild (3)	17 (42.5%)	16 (40.0%)	
Intense (4)	13 (32.5%)	1 (2.5%)	
Very intense (5)	4 (10%)	0 (0.0%)	
Extremely intense (6)	6 (15%)	2 (5.0%)	

**Table 4:** MENQOL scores, DASS-21 scores, and vasomotor symptoms in FS and RJ group at baseline and at the follow-up visit.

Variable	Baseline	Follow-Up Visit	*p*
MENQOL score	111.30 ± 26.17	80.08 ± 28.05	<0.001
Vasomotor domain	17.13 ± 3.19	8.15 ± 3.20	<0.001
Psychosocial domain	26.98 ± 6.52	18.43 ± 7.09	<0.001
Physical domain	53.20 ± 17.25	40.42 ± 18.86	<0.001
Sexual domain	14.00 ± 5.93	12.90 ± 5.57	<0.001
DASS-21 score	20.43 ± 10.70	16.05 ± 10.07	<0.001
Depression score	6.33 ± 3.50	4.6 ± 3.01	<0.001
Anxiety score	6.60 ± 3.76	4.95 ± 3.61	<0.001
Stress score	7.50 ± 5.59	6.48 ± 5.19	<0.001
Hot flushes (n./day)	7.18 ± 2.18	2.85 ± 1.27	<0.001
Hot flushes intensity			
Extremely weak (0)	0 (0.0%)	5 (12.5%)	<0.001
Very weak (1)	0 (0.0%)	8 (20.0%)	
Weak (2)	1 (2.5%)	11 (27.5%)	
Mild (3)	8 (20%)	16 (40%)	
Intense (4)	13 (32.5%)	0 (0.0%)	
Very intense (5)	15 (37.5%)	0 (0.0%)	
Extremely intense (6)	3 (7.5%)	0 (0.0%)	

**Table 5:** Comparison between vasomotor symptoms, MENQOL, and DASS-21 scores after eight weeks of treatment in both groups.

Variable	FS Group	FS and RJ Group	*p*
MENQOL score	100.53 ± 38.56	80.08 ± 28.05	0.008
Vasomotor domain	11.25 ± 4.53	8.15 ± 3.20	0.001
Psychosocial domain	22.60 ± 9.25	18.43 ± 7.09	0.026
Physical domain	50.05 ± 22.50	40.42 ± 18.86	0.041
Sexual domain	14.75 ± 5.87	12.90 ± 5.57	0.153
DASS-21 score	14.05 ± 11.23	16.05 ± 10.07	0.404
Depression score	4.45 ± 3.96	4.6 ± 3.01	0.849
Anxiety score	4.72 ± 4.97	4.95 ± 3.61	0.818
Stress score	4.88 ± 3.43	6.48 ± 5.19	0.108
Hot flushes (n./day)	4.53 ± 3.79	2.85 ± 1.27	0.011
Hot flushes intensity			
Extremely weak (0)	2 (5.0%)	5 (12.5%)	0.465
Very weak (1)	7 (17.5%)	8 (20.0%)	
Weak (2)	12 (30.0%)	11 (27.5%)	
Mild (3)	16 (40.0%)	16 (40.0%)	
Intense (4)	1 (2.5%)	0 (0.0%)	
Very intense (5)	0 (0.0%)	0 (0.0%)	
Extremely intense (6)	2 (5.0%)	0 (0.0%)	

## Data Availability

The raw data supporting the conclusions of this article will be made available by the authors on request.

## Abbreviations

BMI	Body mass index
DASS-21	Depression, Anxiety and Stress Scale
ER	Estrogen receptors
FS	Fermented soy
HDRS	Hamilton Depressive Rating Scale
MENQOL	Menopause-specific quality of life questionnaire
MRJP	Major royal jelly proteins
RJ	Royal jelly
WHO	World Health Organization
